# Green infrastructure site prioritization to improve urban flood resilience in Monterrey and Brussels using a decision support model

**DOI:** 10.1038/s41598-025-94851-z

**Published:** 2025-03-28

**Authors:** Mina Khodadad, Mohsen Sanei, Ismael Aguilar-Barajas, Leopoldo Eduardo Cárdenas-Barrón, Aldo Iván Ramírez-Orozco, Agatino Rizzo, Ahmed Z. Khan

**Affiliations:** 1 Department of Civil, Environmental and Natural Resources Engineering (SBN), Luleå University of Technology, Luleå, Sweden; 2 Building, Architecture & Town Planning (BATir) Department, Université Libre de Bruxelles, Brussels, Belgium; 3 School of Engineering and Sciences, Tecnológico de Monterrey, Monterrey, Mexico; 4 School of Social Sciences and Government, Tecnológico de Monterrey, Monterrey, Mexico

**Keywords:** Green infrastructure, Nature-based solutions, Best management practices, Urban flood resilience index, Decision support tool, Land suitability analysis, Environmental sciences, Natural hazards, Hydrology, Environmental social sciences

## Abstract

Green infrastructure (GI) has been increasingly associated with urban flood resilience as it provides benefits in protecting communities from flood dangers and improving socio-economic capabilities. In order to optimize the GI advantages, it is necessary to engage in strategic prioritizing of implementation areas, considering local conditions. Despite a growing interest in connecting GI and flood resilience, there is still a lack of strategic-oriented GI planning models aimed at enhancing urban flood resilience. This study has introduced the Flood Resilience-based Urban Green Infrastructure Site Priority (FRUGISP) model, which employs a GIS-based multi-criteria assessment to determine the urban regions that should be prioritized for the implementation of GI systems, based on their flood resilience levels. The model was used to map the priority areas in Monterrey, Mexico, and Brussels, Belgium. Despite their distinct features, both cities face flood challenges. The results showed regions of utmost importance based on the flood resilience index and land availability for GI implementation. The model has the potential to be applied to other urban areas grappling with flood issues, providing guidance to decision-makers in selecting high-priority locations for GI projects. This approach can effectively address the difficulties posed by urban floods, ensuring the resilience of urban areas.

## Introduction

Flooding represents the most prevalent kind of catastrophe globally, posing a significant threat to ecological systems, physical infrastructure and structures, and the lives of individuals^1^. In the past two decades, floods have constituted 47% of weather-related disasters, affecting a population of more than 2.1 billion individuals, leading to significant environmental disturbances, and generating economic damages exceeding 1 trillion USD on a worldwide scale^2^. Due to the rising consequences and concerns related to floods, and the inefficiency of conventional flood control strategies and regulations^3^, numerous studies have emphasized the necessity to increase resilience in order to cope with the effects of flooding in urban areas^4–8^.

In the last decade, urban planners have realized the importance of using the ecosystem services provided by nature to deal with urban resilience issues such as urban runoff and flooding^9^. In this regard, green infrastructure (GI) refers to the concept of strategically designed networks of (semi)natural structures that are distinguished by their ability to provide multiple ecosystem services^10^. In planning, GI strategies have emerged to leverage the ability of unpaved, green surfaces to infiltrate water and reduce runoff as well as to retain water and delay peak flow^11^. In environmental engineering and within the context of stormwater management, GI (e.g., rain gardens, permeable pavement, green roofs, etc.) is occasionally used as an alternative for gray infrastructure^8,12^.

The implementation of GI in urban areas has the potential to safeguard both urban residences and infrastructure against the adverse consequences of flooding and extreme storms^13^. Urban GI plays a crucial role in increasing water quality and addressing flood-related issues by mimicking natural hydrological processes such as infiltration enhancement, water storage within the landscape, slowing down the stormwater speed, and reduction of runoff^14^. Additionally, due to its ability to increase urban, societal, and environmental resilience, GI has become a complement to, and in some cases, a temporary replacement for, centralized gray infrastructure^15,16^. Therefore, many cities throughout the globe have adopted GI and its use in spatial planning as a viable technique to improve resilience^17^. However, in order to put GI into operation and achieve optimal efficiency, it is necessary to do a thorough analysis of possible deployment locations^18^.

Earlier research on GI localization based on flood adaptations has mostly concentrated on evaluating flooding risks or estimating reductions in rain/stormwater^18–22^. Consequently, there is a need for optimizing GI locations to increase urban resilience, which includes not only mitigating flooding hazards but also considering pre- and post-flooding procedures^23,24^. Ensuring resilience involves thoroughly assessing natural hazards over their whole life cycle, which includes preemptive measures that may be taken before flooding occurs, such as determining high-density urban areas and optimal spatial configurations for urban land use. On the other hand, immediate rescue efforts and emergency relief programs might be far more efficient if effective response plans were put into place, such as making sure that roads and health amenities are accessible. Likewise, the rapid return of cities to their pre-flood conditions is made possible by recovery measures, such as economic capacity. By considering factors affecting the whole life cycle of flood occurrence in GI localization procedures, this study addresses the mentioned gap in research.

To determine the appropriate location for GI, previous research has taken into account several factors, including land use, hydrology, slope gradient, and soil composition. Nevertheless, the examination of socioeconomic aspects in the implementation of GI practice seems to have been neglected^25^. In order to maximize GI benefits, GI capabilities to provide economic, environmental, and social advantages should be considered^26^. Additionally, to enhance a city’s resilience to floods, it is essential to not only focus on mitigating runoff but also to adopt a comprehensive and integrated approach that incorporates socio-economic and urban infrastructure factors^5^, as the “drivers of vulnerability and adaptive capacity” against flood hazards^6^. As a result, although connecting GI and urban flood resilience is of great interest in recent research^8,23^, there is a shortage of strategic-based GI planning models aimed at improving urban flood resilience, considering vulnerability and adaptive capacity factors. Consequently, the present study attempts to consider a wide range of environmental, infrastructural, and socioeconomic indicators in a model developed to locate GI based on its capacities to enhance urban flood resilience, which will facilitate the creation of comprehensive, strategic development plans and serve as a valuable point of reference for all parties involved and stakeholders^24^.

According to Zuniga-Teran, et al.^27^, the performance of GI with regard to urban resilience is a complicated issue that needs to be further assessed. Additionally, conducting more thorough quantitative research with a range of features and parameters is advised to address inclusive relevant aspects of GI and urban flood resilience^8^. This research contributes to these issues by developing a Flood Resilience-based Urban Green Infrastructure Site Priority (FRUGISP) model, which serves to identify priority locations for GI implementations, as a crucial phase in determining the future direction of sustainable and resilient urban development^18^. The structure of the created GIS-MCDA (Geographic Information System; Multi-Criteria Decision Approach) model is akin to land suitability analysis and it considers various infrastructural, socioeconomic, and environmental aspects in order to determine the priority for implementing GI inside urban areas.

The performance of the model is established in two case-study regions with different characteristics but common urban flood threats (Monterrey City in Mexico and Brussels-Capital Region in Belgium), verifying that the model could be used in other cities as well. Both study areas lack spatial mapping for GI implementations to address their existing water security challenges, including flooding^28^. Our model fills this local gap as well, showing the high-priority areas for GI applications to enhance urban flood resilience. FRUGISP has the potential to be used as a tool for facilitating future planning for urban GI deployments, providing the chance to create environment-conscious, resilient, and sustainable urban environments^29–31^.

The article is organized as follows: the second section outlines the methodology utilized for data collection and analysis generation. Section three presents the analytical results for each city, followed by section four, which discusses the most significant outcomes of the analysis within their local context. The concluding section encapsulates the research outcomes and identifies certain limitations and avenues for future research.

## Methodology

This research uses a spatial analysis framework which is the most used methodology in the recent studies connecting GI and urban flood resilience according to Khodadad, et al.^8^. Accordingly, the “Flood Resilience-based Urban Green Infrastructure Site Priority” (FRUGISP) model is developed, using a GIS-based MCDA methodology, to identify the priority areas that require urban green infrastructure adaptations to increase flood resilience in Monterrey City (MTY), Mexico, and Brussels-Capital Region (BCR), Belgium. The FRUGISP model is built upon the land suitability analysis (LSA) approach, which is used frequently in optimizing land use management. The main objective of LSA is to identify the least and most suitable areas for the placement of a particular goal, such as prospective land use^32^. In LSA methodology, the landscape is perceived as the outcome of intricate connections between multiple aspects, such as environmental, social, and economic^17^. The objective of LSA is to enhance the management of environmental assets while maximizing the advantages derived from other sectors and considering the primary components of the natural environment, including the social values of the region together with the biological mechanisms and the physical characteristics^17^.

To determine the optimal sites for GI interventions, it is imperative to concurrently evaluate multiple variables. As a result, the Multi-Criteria Decision Approach^33^ and Geographic Information System were combined in this study. The GIS-based MCDA methodology^34^ is a prominent method employed by multiple fields to assess site suitability for the optimal application of diverse objectives^18^. The MCDA seems to be advantageous as it enables the examination of many options, taking into account the different objectives and opposing preferences^35^. It is widely employed for conducting land use suitability simulations, focusing on generating a single index assessment by merging multiple data layers of various criteria^36^. Consequently, it has been extensively employed to identify suitable sites for GI implementations according to the unique features of each site^17^, optimizing the advantages of GI^15,37^, and encouraging the adoption of particular GI forms^18^.

**Fig. 1:**
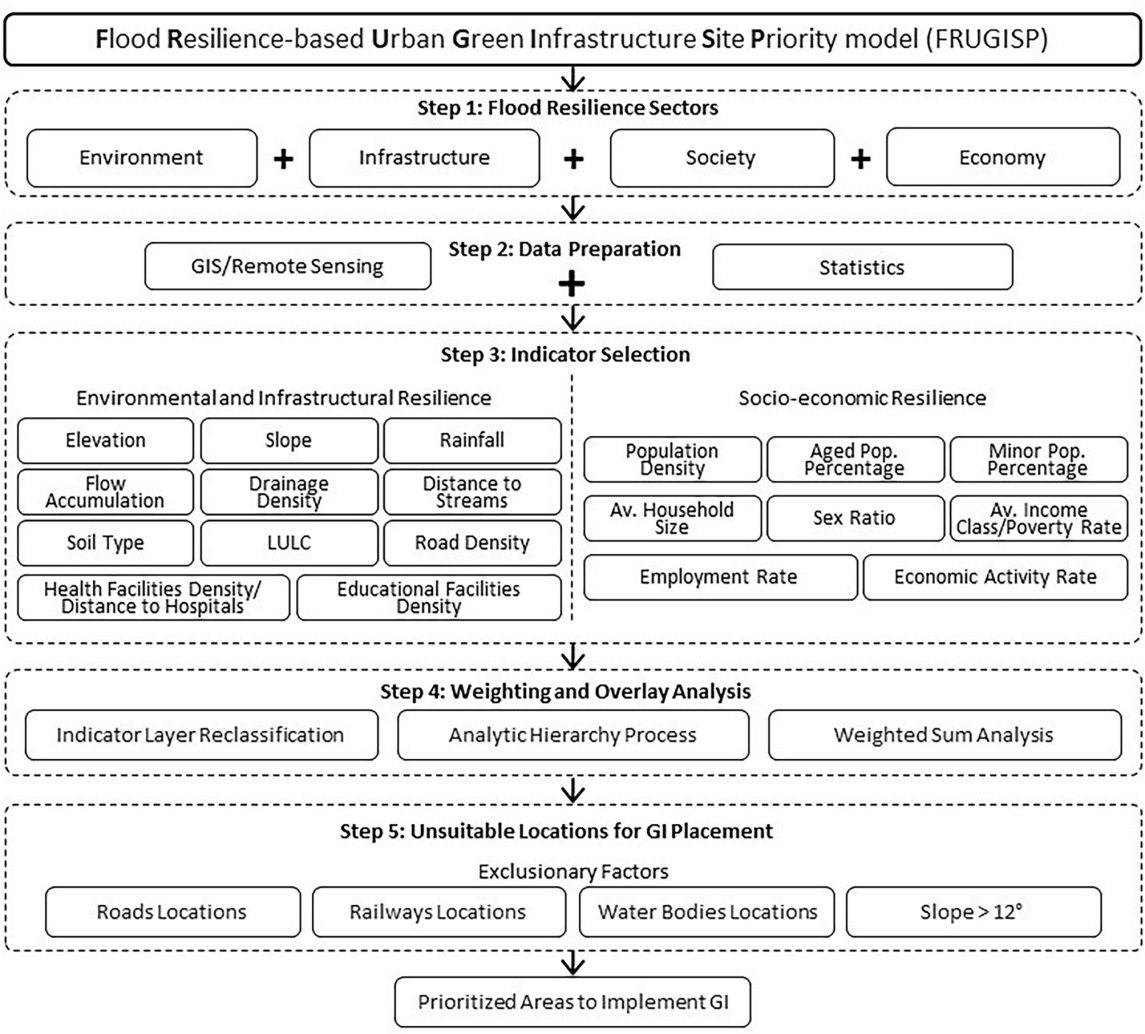
Flood resilience-based urban green infrastructure site priority (FRUGISP) model.

The model (Fig. 1) was developed based on the selection and integration of many different indicators to assess and examine regions by combining diverse criteria and integrating multiple layers of geospatial data to create a comprehensive single index assessment. The objective of the model is to identify spatial priorities for the GI implementations as a solution to the urban flood resilience challenges that the areas are now experiencing and require attention. The model suggests that to maximize and strengthen the significance and advantages of GI in addressing the significant local problem of urban flooding, implementation of GI should be prioritized in regions where urban flood resilience is lacking. Likewise, based on some exclusionary criteria, the locations where GI implementations are not physically advised or possible are excluded from the analysis. Consequently, based on the flood resilience assessment and the exclusionary factors, the most prioritized regions in need of GI localization will be determined by the model in the study areas.

To facilitate the evaluation of urban flood resilience, the present study has created a quantitative framework getting references from the recent models that have been developed for this reason^6,24,38,39^. In this regard, first, a set of indicators, taking into account four primary sectors of resilience was developed to assess urban flood resilience. Second, the Analytic Hierarchy Process (AHP) was used to weigh the standardized indicator parameters. Then, the overlay analysis was conducted to demonstrate the results by determining the levels of flood resilience. This process was followed by excluding some areas from the study areas, based on exclusionary criteria, giving us the ultimate outcomes of the GI priority analysis.

### Study areas

**Fig. 2:**
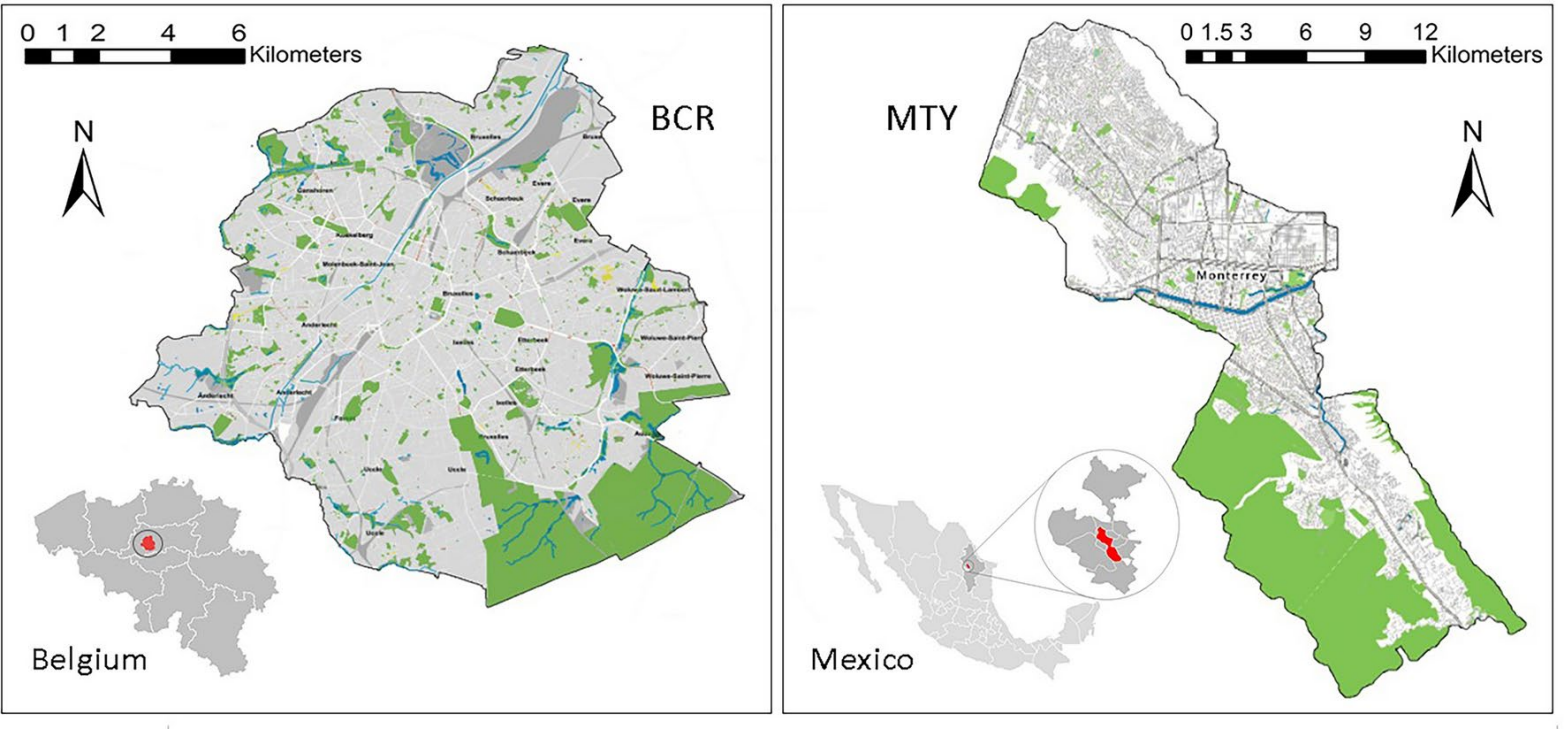
The study areas and their open green/blue spaces: BCR (left) and MTY (right), adapted from public domains: https://environment.brussels/ and https://www.inegi.org.mx/.

This study looks at the Brussels-Capital Region in Belgium and Monterrey City in Mexico. Both cities suffer from serious water security challenges^28,40,41^, including flooding^42,43^. Figure 2 and Table 1 present the location and general outlines of the selected areas.

**Table 1:** The general characteristics of the study areas.

Characteristics	Monterrey city	Brussels-capital region
Area (km^2^)	324.4^44^	161.4^45^
Population (million)	1.143^44^	1.209^46^
Average rainfall/year (mm)	590.8^47^	837.1^45^
Average annual temperature (°C)	22.3^47^	10^45^
Climatic condition	Warm and temperate (Cfa)^48^	Mild temperate, fully humid, warm summer (Cfb)^49^
Administrative level	Municipality^50^	Capital region^42^

### Urban flood resilience quantitative assessment and indicators

Given the complexity of the topic of flood resilience, we adopted the approach of utilizing the sectors of resilience as delineated in the flood resilience indices produced by^6,24,38,39^, as a guide for selecting the variables that would be employed to assess the level of resilience. The sectors encompass the domains of environment, society, economics, and infrastructure. The mentioned studies have confirmed the correlation between flood resilience and the environmental, infrastructural, social, and economic sectors. As a result, this study has developed a comprehensive index framework for assessing urban flood resilience and has identified relevant indicators within each of the four sectors.

The concept of environmental resilience pertains to the capacity of a natural environment to endure, adapt, and recover its original structure through inherent resilience mechanisms in the face of disturbances^51^. The concept of infrastructural resilience pertains to the capacity of infrastructure systems to absorb damages, endure catastrophic events, and recover their regular operations subsequent to the catastrophe^52^. Furthermore, the concept of social/economic resilience refers to the potential of urban systems to sustain their regular social order/economic functioning in the presence of external disruptions and calamities^24,53^. Following the approach of^6,24,38,39^, to include all the resilience sectors with their relative degree of importance, the quantitative model of urban flood resilience is regarded as:

1 $$FR={w}_{1}\times EnR+{w}_{2}\times IR+{w}_{3}\times SR+{w}_{4}\times EcR$$

where *FR* represents flood resilience; *EnR*, *IR*, *SR*, and *EcR* stand for environmental resilience, infrastructural resilience, social resilience, and economic resilience; and *w*_*1*_*, w*_*2*_*, w*_*3*_*, and w*_*4*_ are their assigned weights, respectively (see Section. “Weight assignment using analytic hierarchy process” and Supplementary Table S1 for weightings).

The indicators related to social and economic sectors are all derived from statistical data, while the indicators for the infrastructure and environment are all derived from GIS and remote sensing data. The indicators were chosen based on a literature review that considered several international research on the subject. Table 2 includes the list of the most frequent variables found in the literature, explaining the selection basis for each. Only the factors for which data was publicly available or could be constructed were listed. Also, two related factors might be adopted to address a similar issue in the two cities (e.g., average income class for BCR and poverty rate for MTY are used to show the level of household economic development). This adoption was done because of differences in the locally available data. A total of 19 factors were ultimately chosen for inclusion in the flood resilience index for each city.

**Table 2:** Urban flood resilience indicators and their selection basis.

Criterion layer	Indicator layer	Selection basis
Environment	Elevation	The stress of urban storm flood systems is influenced by elevation, with low-lying regions being more susceptible to precipitation and damage caused by flooding^4,54^
	Slope	The slope has a crucial role in determining the velocity of current floods^55^
	Rainfall	Precipitation has a significant role in the occurrence of flood disasters^39,56^
	Flow accumulation	Flow accumulation is well recognized as a hydro-geomorphic factor that contributes to the assessment of flood disasters. Greater values are indicative of a greater possibility for runoff^57^
	Drainage density	The higher the density of the drainage network, the greater the susceptibility of urban areas to flood impacts^58^
	Distance to streams	There is a positive correlation between the proximity of a region to water lines and its susceptibility to flooding^59^
	Land use land cover (LULC)	In the occurrence of a flood, diverse land use categories exhibit distinct levels of flood damage and varying degrees of vulnerability. In contrast to green areas, impermeable ground exhibits reduced capacity for water absorption and heightened susceptibility to floods^39,60^
	Soil hydrologic group	The categorization of soil hydrologic groups plays a crucial role in assessing its capacity to support the process of runoff infiltration, particularly in the context of flood management^18^
Infrastructure	Road density	The density of roads has a significant impact on the efficiency of evacuating individuals during flood catastrophes, hence contributing to the enhancement of resilience^57,61^
	Health facilities density/distance to hospitals	Health facilities have a crucial role in determining the level of community vulnerability to the impacts of disasters like floods^4^. The presence of additional or proximate healthcare facilities inside a community enhances the community’s resilience to natural catastrophes, such as floods
	Educational facilities density	Educational establishments within a community can serve as shelters or evacuation hubs during times of emergency or catastrophe scenarios^54^. The presence of a greater number of educational institutions is indicative of the provision of optimal shelter services within a community^4^
Society	Population density	There is a positive correlation between population density and the severity of damage resulting from flood catastrophes^62^
	Aged population percentage	Elderly people are considered vulnerable in flood resilience evaluation^6,24^
	Minor population percentage	Minors are considered vulnerable in flood resilience evaluation^24,63^
	Household size	It is anticipated that a region with a large household size will have poor flood resistance^5,64^
	Sex ratio	The vulnerability of a place to natural catastrophes, such as floods, is influenced by its sex ratio, as women are commonly believed to be at a heightened risk within the vulnerable population^57,64^
Economy	Average income class/poverty rate	Typically, regions with lower economic development have a greater vulnerability to floods^61^. A higher income level and a lower poverty rate contribute to the increased resilience to flood events^5,6^
	Economic activity rate	Economic resilience is affected by the rate of economic activity^65^
	Employment rate	The employment rate is a significant determinant of societal stability. There exists a positive correlation between the extent of damage resulting from a flood catastrophe and a decrease in the employment rate^57,64^

### Identification of unsuitable GI implementation sites

In order to define the suitable sites for GI adaptations, based on analysis of relevant literature, four exclusionary criteria have been established (Table 3) as indicators of the unsuitability of certain locations for the implementation since they should be excluded from the potential GI sites^66^. In regard to the present study, areas that are not accessible for GI applications, such as railroads, roadways, and avenues, as well as those that are currently occupied by surface water are considered unsuitable. This has been done as, to avoid additional implementation costs, GI is mostly set up in spaces that are already available for implementation. So, in line with previous studies (e.g.^18^), we decided to exclude all parcels of land used by transit systems (although in cases with low availability of free space, limited GI applications can be done within these areas). Also, places already occupied by surface water mostly do not offer additional runoff retaining capacity by GI installations that can be used to enhance urban flood resilience. Therefore, water bodies have been eliminated from the analysis as well.

**Table 3:** Exclusionary factors used to delineate unsuitable locations.

Category	Reason	Exclusion criteria
Transportation network	Unavailability of land	The land areas that are categorized as roads or streets
	Unavailability of land	The land areas that are categorized as railway zones
Watershed features	Unavailability of land	All surfaces that fall under the classification of water bodies, such as ponds, river basins, and waterways
Topography	Impact on the flow velocity of surface water	Slopes exceeding 12°

Additionally, locations characterized by sloping ground are considered unsuitable due to their impact on the velocity of the flow of surface water^67^. Different grades of slope steepness might be regarded as suitable for GI installations, based on the type of GI being used, but in accordance with the suggestion put out by McFarland, et al.^68^, we decided to eliminate the regions characterized by a slope over 12°. Unsuitable sites were excluded from the final urban flood resilience analysis results.

### Weight assignment using analytic hierarchy process

The Analytic Hierarchy Process (AHP) is a multi-level method of weight analysis that was introduced by T. L. Saaty, an American operational research scientist, during the late 1970s^69^. The methodology relies on expert knowledge and is employed in the assessment of land suitability, to ascertain the division of weighting values^69^. This technique is a multi-criteria decision-making procedure that is well-suited for addressing intricate decision-making scenarios^70^. AHP is explained as “the most reliable and flexible multi-criteria analysis method^71^” and a literature review by De Brito and Evers^72^ found that, in the field of flood management, AHP is the most popular MCDM method. To conduct pair-wise comparisons and perform weight and consistency ratio (CR) computations in this study, the AHP Spreadsheet developed by Pyzdek^73^ was utilized. The relative relevance value of each element in the pairwise comparison matrix was determined by evaluating each item with all other variables using a scale for comparing factors, as suggested by Saaty^74^. This scale varied from 1 to 9, with 1 indicating equal significance and 9 indicating extreme significance (see Supplementary Table S2).

Fifteen experts were primarily asked to participate in the AHP weight assignment of the indicators. They were either university professors or researchers in the fields of urban water/flood management and urban planning, with high educational levels (PhD or PhD researcher). All experts were either Belgian or Mexican, working in higher educational institutions, to ensure a high level of knowledge on local contexts and challenges. Two rounds of emails were sent to the experts asking for their participation in our ranking. Ten experts accepted and did the pairwise comparisons on AHP Excel templates. It was also asked to report their knowledge about the urban flood resilience analysis based on their assumptions. Three judgments were eliminated as the experts reported ‘limited’ knowledge of the field.

In total, the opinions of seven experts were used to rank the weighting of indicators (judgment matrices are included in the supplementary data). Although this is in line with several previous recent studies where similar numbers of experts were used for AHP ranking^39,75–79^, higher numbers would be beneficial to minimize the subjectivity of the experts’ judgments. Accordingly, our sample size should be noticed when interpreting the outcomes of this exploratory part of our research. The final expert group included four Mexicans and three Belgians from Tecnológico de Monterrey, Université libre de Bruxelles, and Vrije Universiteit Brussel institutions. More information about the experts’ profiles can be found in Supplementary Table S3. The group judgment on the weighting of indicators was achieved by aggregation of individual judgment (AIJ) method^80^, using round-off values of geometric means for each pairwise comparison^76^. The weight for each indicator was then obtained by normalizing the score matrix.

Since in practice the expression and the statement of the decision maker include some fuzziness and might cause inconsistencies in the matrix, a verification of consistency was carried out by calculating the CR, which represented the quality and accuracy of the pair-wise comparisons^81^. When 0 $\le$ CR $\le$ 0.1, it signifies that the matrix has successfully undergone a consistency test, hence indicating that the assigned weights for each indication are considered reasonable^69^. If not, the matrix must be modified until a suitable level of consistency is reached (the experts were asked to check the CR values and if needed modify their judgments to reach consistency). The Flood Resilience Index (FRI) was calculated by combining the weights and corresponding normalized resilience values for each indicator through the use of Eq. (2):

2 $$FRI={\sum }_{i=1}^{n}\omega i\times \partial i$$

where *FRI* stands for the flood resilience index, *n* signifies the number of criteria, $\omega$_*i*_ denotes each indicator’s weight, and $\partial$_*i*_ denotes the indicator’s normalized value at each location (pixel on the raster map).

### Normalization of the parameters

During the evaluation process, it is important to conduct an analysis of indicator parameters. Due to the disparate units and dimensions of indicators, the process of calculating them in relation to one another can be complex. To avoid the impact of different indicator units and the vast disparity in numerical magnitude, every indicator needs to be considered dimensionless to overcome the challenge of incomparability across indicators. Consequently, all indicators were rendered dimensionless and normalized through reclassifying the quantitative values and dedicating numerical numbers to the qualitative data, e.g., the sub-indicators of soil type and LULC, depending on their contribution and impact on the flood resilience and GI priority. This has been done mostly by natural breaks within ArcMap (please see the next section), which are based on Jenks Natural Breaks algorithm^82^. In a few cases where natural breaks could not provide optimal results based on the research aims, manual breaks were used. For instance, because of the existing mountainous areas within the MTY city border, we chose not to use natural breaks for the MTY slope map, as most urban areas would be considered with the same level of flood resilience, which was not accurate for our analysis.

The indicator values for the reclassification are associated with the flood resilience or GI priority rating flood resilience or GI priority rank (depending on the objective of the map), which ranges from 1 to 10 showing the minimum and maximum resilience/priority values. If the difference between minimum and maximum original values was not significant, we did not use all 1–10 classes. The rainfall map of BCR is an example where only four classes were made with scores from 4 to 7. A value of 0 was also assigned to the sub-indicators or values that should not be included in the analysis, eliminating them from the overlay (weighted sum) analysis (e.g., water in LULC).

### Application of FRUGISP model in GIS

To use the GIS-MCDA method, it is necessary to ensure that all criterion data are appropriately recorded in a geospatial format. Consequently, all relevant variables were represented in ArcMap 10.8.1^83^ for each specific study area. All indications under consideration were provided in a grid raster format to facilitate the implementation procedure. The unit of analysis for socioeconomic indicators was Basic Geostatistical Area for MTY (AGEB in Spanish) and neighborhood for BCR, to have a sufficient level of detail in the analysis. The first step in data preparation was converting the data into spatial layers and assigning the appropriate spatial reference system (Mexico_ITRF_2008_LCC for MTY and Belge_Lambert_2008 for BCR). Additionally, the layers were resampled to match the spatial resolutions of the DEM layers, which were 15 m for MTY and 20 m for BCR. This process facilitated the alignment of grid cells over many raster layers.

The indicator raster layers and weights established in the AHP for each indicator were used to develop the FRUGISP model (see Fig. 1). The indicator layers were overlayed using the “weighted sum” analysis tool of ArcMap, creating composite flood resilience maps (environmental, infrastructural, social, economic, and overall FRI), and GI priority maps (eliminating places deemed inappropriate for GI deployment). Consequently, a total of six distinct resilience/priority maps were acquired for each case study region.

The raster pixel values for each spatial layer are divided into ten resilience classes, with red color showing the minimum resilience class (1 of 10) and blue illustrating the maximum resilience level (10 of 10). Same color ramps are used to show resilience values for maps with less than 10 value classes (e.g., soil and LULC maps). The gray color is used for values that are excluded from resilience classes (i.e., water in LULC). In some cases (e.g., sex ratio), a single color is used for two value classes, showing that a similar resilience class is assigned to both value ranges. For easier visual identification between indicator maps and combined spatial layers, a different color ramp is used for the resilience sectors maps, overall FRI maps, and GI priority maps, with red showing the minimum resilience/maximum priority and green illustrating maximum resilience/minimum priority.

### Data collection

To guarantee that the final outcome can be replicated, confirmed, and enhanced with additional data by stakeholders, all data utilized were acquired from open databases. The BCR soil map shapefile was the only exception which was acquired from the BONAT project team^84^. GIS and remote sensing data are used for the environment and infrastructure sectors, while statistics data are used for the socioeconomic components. Table 4 lists the data categories and sources.

**Table 4:** Data types and sources of urban flood resilience and exclusionary criteria.

Criteria	Data type		Data details		Data source	
	MTY	BCR	MTY	BCR	MTY	BCR
Elevation	CEM 3.0 Nacional	DTM	15 m, 2013	20 m, 2022	INEGI	geo.be
Slope	Raster data		15 m	20 m	Elaborated*	Elaborated
Flow accumulation	Raster data		15 m	20 m	Elaborated	Elaborated
Drainage density	Raster data		15 m	20 m	Elaborated	Elaborated
Distance to streams	Raster data		15 m	20 m	Elaborated	Elaborated
Average annual rainfall intensity	Attribute data	Attribute/ Vector data	1951–2010	1991–2020	CONAGUA	The Royal Meteorological Institute
LULC	Vector data		2013	2016	Gobierno de Monterrey	Urban Atlas 2012; geo.be
Soil type	Vector data		2013	2008	INEGI	BONAT
Population density	Attribute data	Vector data	2020	2021	INEGI	bisa.brussels
Aged population percentage	Attribute data	Vector data	Over 60 y/o, 2020	Over 65 y/o, 2021	INEGI	bisa.brussels
Minor population percentage	Attribute data	Vector data	Under 14 y/o, 2020	Under 17 y/o, 2021	INEGI	bisa.brussels
Household size	Attribute data	Vector data	2020	2021	INEGI	bisa.brussels
Sex ratio	Attribute data	Vector data	2020	2021	INEGI	bisa.brussels
Average income class/poverty rate	Attribute data	Vector data	2015	2019	CONEVAL	bisa.brussels
Economic activity rate	Attribute data	Vector data	2022	2020	INEGI	bisa.brussels
Employment rate	Attribute data	Vector data	2022	2020	INEGI	bisa.brussels
Health facilities/ hospitals	Vector data		2022	2018	INEGI	Brussels mobility
Educational facilities	Vector data		2022	2018	INEGI	Brussels mobility
Roads	Vector data		2013	2016	Gobierno de Monterrey	Urban Atlas 2012; geo.be
Railways	Vector data		2013	2016	Gobierno de Monterrey	Urban Atlas 2012; geo.be
Water bodies	Vector data		2013	2016	Gobierno de Monterrey	Urban Atlas 2012; geo.be

* The data is created from DEM in ArcMap.

## Results

### Index indicator weights

Supplementary Table S1 indicates the assigned weightings to the FRI indicators and sectors. The CR values for five judgment matrices constructed for AHP weightings of different flood resilience sectors are between 0.000 and 0.038 (see Tables S4–S8), validating the reasonability of the judgments (0 ≤ CR ≤ 0.1). Using the assigned weights, multiple spatial layers of FRI are combined, shaping the maps of each flood resilience sector together with the overall FRI map for each study area. Eliminating the exclusionary criteria from the FRI maps, the final outcomes of the FRUGISP model are created showing the priority areas suitable for GI implementations in the study areas. The results of the model are described below.

### Monterrey city

#### Flood resilience sectors and indicators

**Fig. 3:**
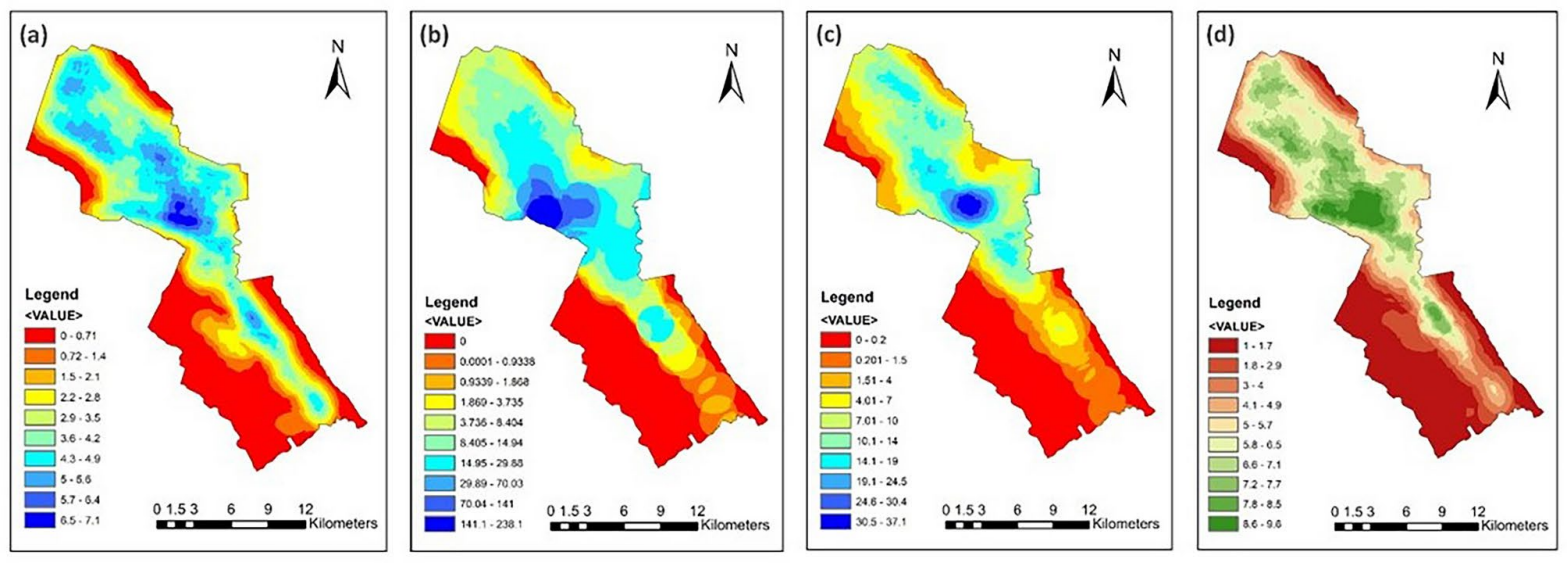
MTY: (**a**) Environmental Resilience Index; (**b**) Infrastructural Resilience Index; (**c**) Social Resilience Index; (**d**) Economic Resilience Index. Maps created by ArcMap 10.8.1^83^.

The results of the FRUGISP model analysis for MTY are described in this section. Each flood resilience sector is shown in Fig. 3. Considering EnFRI (Fig. 3a and Supplementary Fig. S1), the presence of mountains on the western and eastern sides of MTY is marked by high elevations, steep slopes, and forest land cover. Urban development areas are mostly located on soil with fine texture, which represents clay soil type with the worst infiltration rate according to INEGI’s three soil textures (thick, sandy; medium, silt; fine, clay). Rainfall intensity is diverse in the area, with southern parts marked with higher intensities. Southern parts of the city also have greater drainage density quantities, showing a higher density of streams in these areas. These indicators seem to have the main contribution to low overall EnFRI in the southern urban areas. In the northern parts, the areas with higher drainage density and closer distance to streams are marked by low EnFRI.

The lowest infrastructural FRI (IFRI; Fig. 3b and Supplementary Fig. S2) values are shown in mountain areas where lower urban development, and consequently lower density of roads and health/educational facilities, is present. On the other hand, the central parts of the city present higher road health/educational facilities density. This part of the city includes the historical heart characterized by dense road networks, together with major commercial, administrative, and attraction hubs, and many health and educational centers. Therefore, the IFRI is maximum mostly in these areas.

**Fig. 4:**
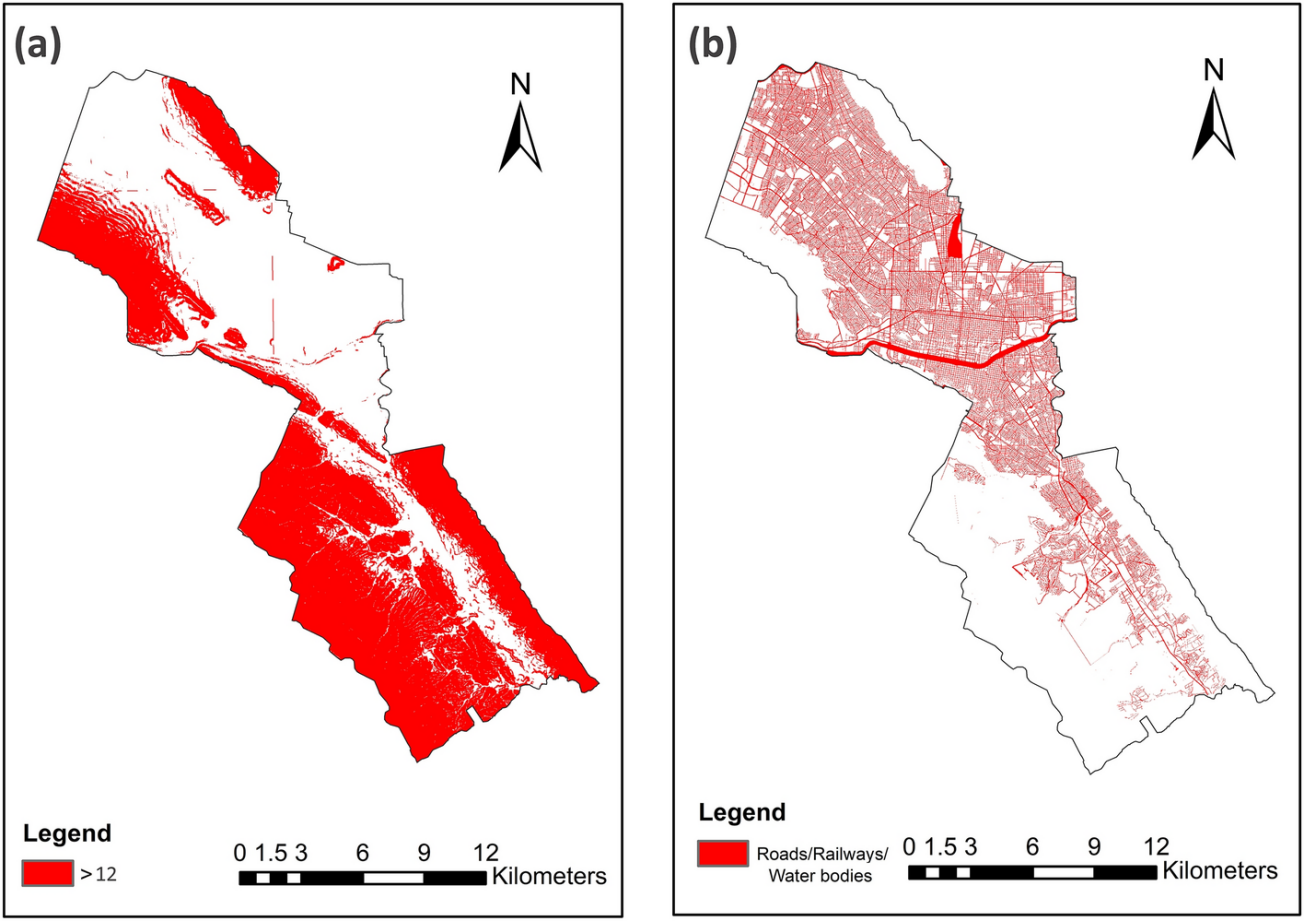
MTY: GI unsuitable locations: (**a**) Slope greater than 12°; (**b**) Roads, railways, and water bodies. Maps created by ArcMap 10.8.1^83^.

Social FRI (SFRI), is depicted in Fig. 3c and Supplementary Fig. S3. White areas illustrate the parcels with no available data or outside urban development areas. In case the data is not available for an AGEB (unit of analysis), the area is excluded from the SFRI or the overall FRI analysis (see Fig. 4). SFRI layer represents lower resilience levels for mostly the northern and some central parts of the city. This is mainly due to higher population density, higher minor population rate, and bigger household size in this region.

The economic FRI (EcFRI; Fig. 3d and Supplementary Fig. S4) is the last of four FRI sectors, which displays relatively comparable values to SFRI, showing a high level of interdependencies of socio-economic factors in the region. As shown, similar to SFRI, lower flood resilience indices are seen in the northern and some central areas of the municipality, which are among the most populated areas of the city, as well. However, some southern parcels also have low levels of EcFRI (low employment and economic activity rates and high rate of poverty), unlike their SFRI and the population density rate. Most AGEBs in the area have a rate of poverty below 18 percent, while in a few areas, more than half of the population is considered poor. Employment and economic activity rate indices show relatively similar trends as the indicators are highly related to each other. These indicators are considered maximum, where no person lives in an AGEB. This is due to the fact that areas with zero populations are considered resilient against natural disasters.

#### GI unsuitable locations

To construct the final GI priority index for the city, four exclusionary factors were determined, namely the location of roads, railways, and water bodies (e.g., streams or river beds), and the areas with slopes greater than 12°. The first three indicators are mutually displayed in Fig. 4b, and the slope exclusion is depicted in Fig. 4a. The unavailable land for GI implementation is highlighted in red. As seen, almost half of the whole city area (153.13 km^2^) is excluded as it has slope degrees greater than 12°. This includes mostly the mountains and the foothills that are mostly located in the southern parts, as well as the Santa Catarina riverbed in the center. The riverbed is also marked in Fig. 4b, along with a dense network of roads, mostly placed on the northern side, where urban development is widened.

#### Overall FRI and GI site prioritization

**Fig. 5:**
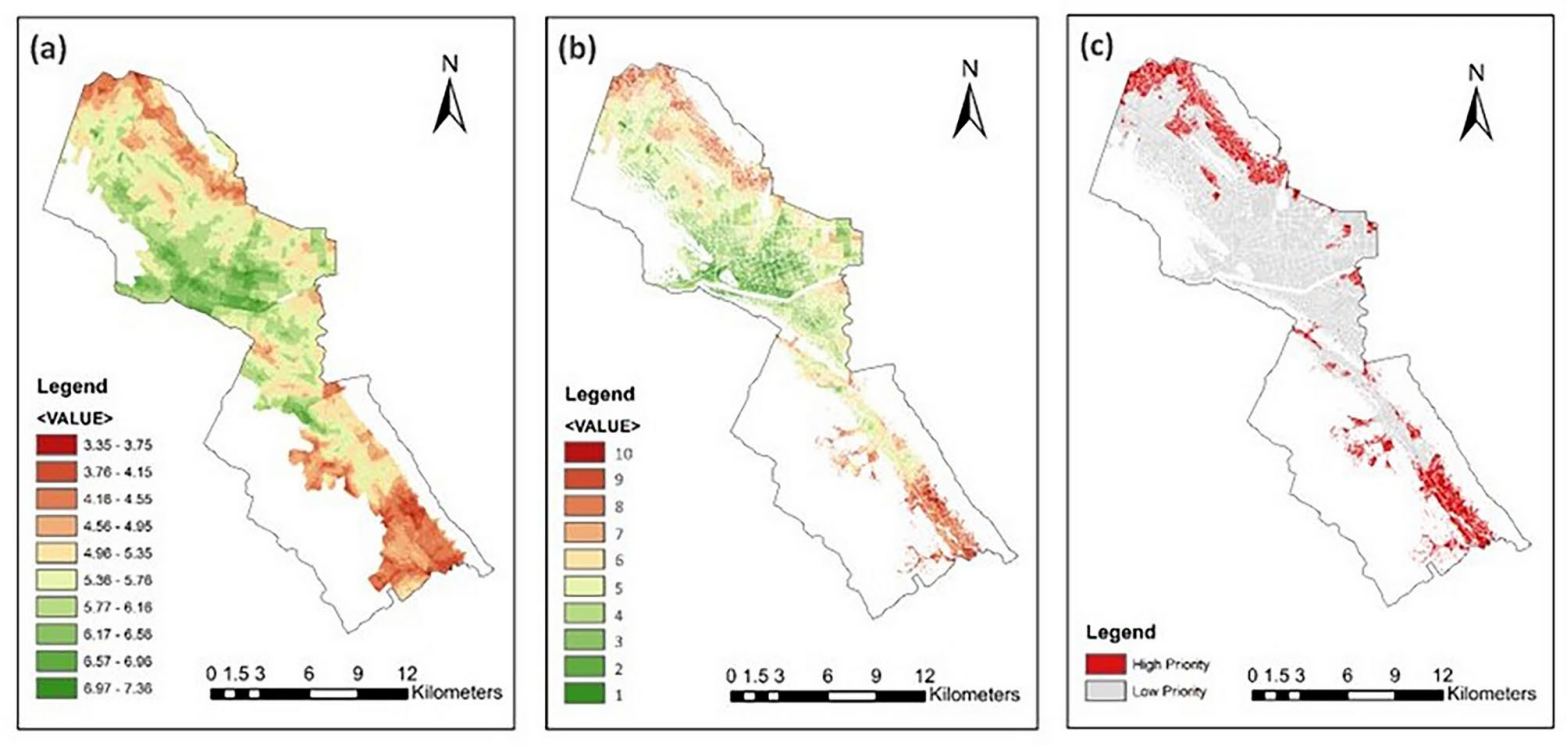
MTY: (**a**) Overall flood resilience index; (**b**) Overall GI implementation priority index; (**c**) Areas with more/less than moderate flood resilience index (high priority: FRI < 5). Maps created by ArcMap 10.8.1^83^.

The final outcomes of the FRUGISP model analysis for MTY are displayed in Fig. 5. Figure 5a depicts the spatial distribution of FRI in the city, with the western-central areas having the highest indices and the southern and northeastern parts associated with low FRI values (FRI_min_ = 3.35, FRI_max_ = 7.36). Low elevation, slope, and soil infiltration capacity, along with closeness to streams, dense population, low infrastructure density, and high poverty are among the main reasons making the overall FRI of the northeastern parts very low. Likewise, the critical causal factors for low FRI values in the southern parts are high rainfall and drainage density, high minor population rate, low employment and economic activity, and low density of urban infrastructure, especially health and educational facilities. On the other hand, high flood resilience indices in the western-central parts are due to low rainfall intensity and drainage density, high density of all urban infrastructure, low general/minor population density and average household size, and high economic resilience.

Excluding the four exclusionary factors (Fig. 4), Fig. 5b shows the GI localization priority index of the study area. In total, almost half (103.27 km^2^) of the total 209.21 km^2^ areas with FRI values were excluded using the exclusionary factors. Using the natural breaks classification of the FRI values, the GI localization priority is divided into 10 levels (the lower the FRI, the higher the GI implementation priority index). Ultimately, the spatial distribution of areas where FRI is below the moderate level (i.e., FRI < 5; areas with a resilience value below average) is depicted in Fig. 5c. This includes almost a quarter (24.52%) of the total areas that received FRI and where GI implementation is possible. In other words, 25.98 km^2^ of the total 105.94 km^2^ has high priority for GI localization in order to minimize the overall flood resilience in Monterrey.

## Brussels-capital region

### Flood resilience sectors and indicators

**Fig. 6:**
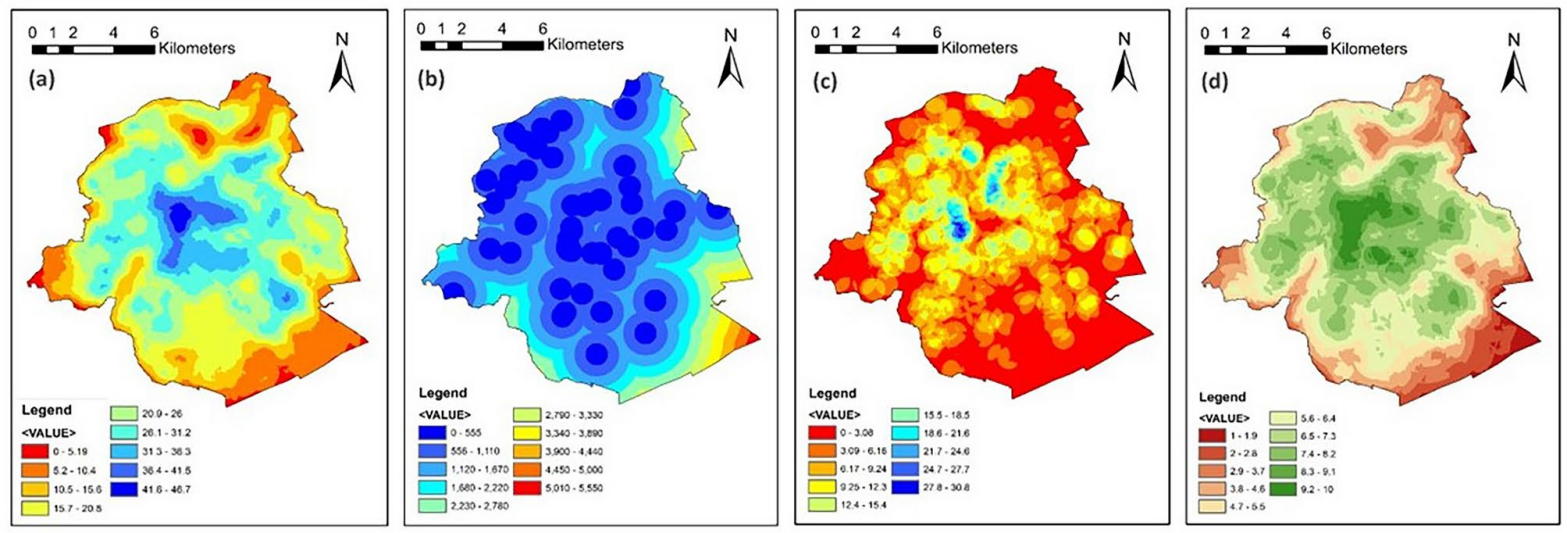
BCR: (**a**) Environmental Resilience Index; (**b**) Infrastructural Resilience Index; (**c**) Social Resilience Index; (**d**) Economic Resilience Index. Maps created by ArcMap 10.8.1^83^.

The same analysis is done for BCR. Figure 6 depicts the overall assessment of four resilience index sectors. As can be seen, EnFRI (Fig. 6a and Supplementary Fig. S5) has the lowest levels mostly in a section stretched from north to west-south of the region, which is characterized by lower elevation, a high proportion of very dense urban development, soil classes with worst infiltration rates, and high drainage density. As the rainfall intensity does not differ a lot in various parts of the region, only four levels of resilience are assigned to the classes ranging from 4 to 7. Therefore, rainfall does not play a key role in differentiating the flood resilience index in BCR.

The overall IFRI is depicted in Fig. 6b and Supplementary Fig. S6. As the information regarding the spatial distribution of health facilities in BCR includes only the location of hospitals and no other related facility (e.g., clinics), a distance-to-locations analysis is performed for the case of BCR (instead of density analysis which makes more sense where a high number of facilities are analyzed). The IFRI levels in the region show higher degrees of resilience in the central parts, as opposed to the peripheral areas. It is mainly because the urban development in these areas is very dense with high levels of road and facility densities. The presence of forests in the southeastern parts is the reason for being classified with the lowest levels of IFRI, as no/very few infrastructural elements are located in this area.

The next resilience sector in the FRUGISP model is the social sector. Five different layers are combined to shape the SFRI map of BCR (Figs. 6c and Supplementary Fig. S7). The areas with no available data are colored white and are excluded from the overlay analysis. It seems that the population percentage of minors, average size of households, and population density have the greatest impact on the overall SFRI. Although bordering districts have less population density, they involve relatively higher average household sizes and elderly population rates. Also, in most neighborhoods, the population of women surpasses the number of male inhabitants, which is higher mostly in central parts. In general, neighborhoods located on the northwest side of BCR are less socially resilient to urban flooding.

The outcomes of the analysis for the economic sector, as the last analyzed flood resilience dimension, show higher levels of resilience mostly in central neighborhoods (Fig. 6d and Supplementary Fig. S8). Due to the availability of data and higher economic development conditions of the region, for BCR, the average income class for each neighborhood is used instead of the poverty rate. Unlike MTY, where the spatial distributions of the economic and social resilience indices were relatively similar in the city, here the SFRI map shows more similarity to IFRI rather than EcFRI, as both SFRI and IFRI have higher values in central areas of the region. Among the variables, the employment and economic activity rates seem to be more related in comparison to the average income class distribution.

#### GI unsuitable locations

**Fig. 7:**
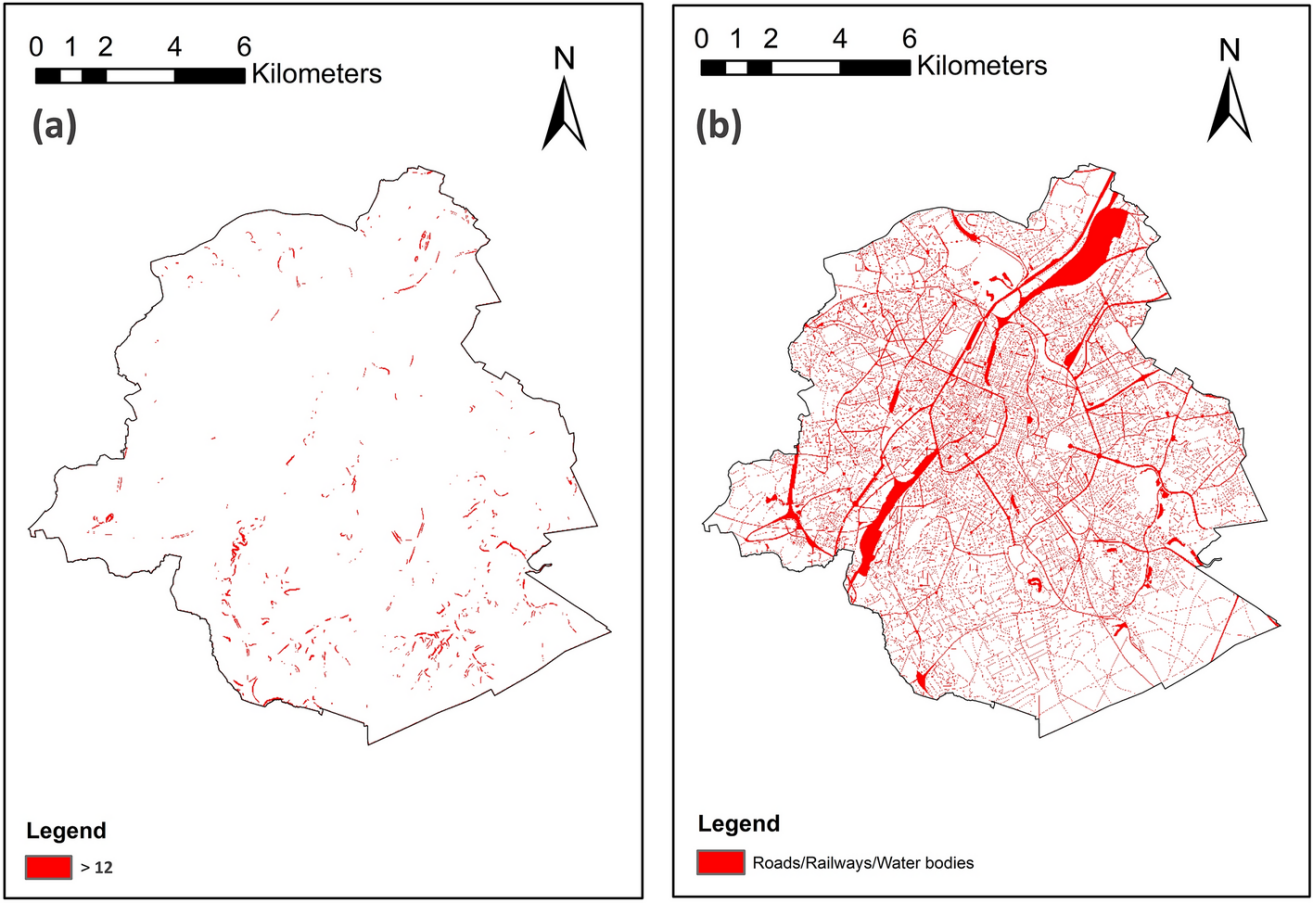
BCR: GI unsuitable locations: (**a**) Slope more than 12°; (**b**) Roads, railways, and water bodies. Maps created by ArcMap 10.8.1^83^.

As seen in Fig. 7, unlike MTY, most BCR areas fall in the acceptable range regarding the slope conditions. This shows that BCR does not have many parts with steep slopes. Accordingly, only less than 2 percent (1.86%; 3.03 km^2^) of the total area of around 160 km^2^ was excluded by the slope limiting condition for GI implementations (Fig. 7a). Other exclusionary items (roads, railways, and water bodies) are shown in Fig. 7b, marking a comparatively greater part of the city (14.82%; 24.07 km^2^) as inappropriate for GI localizations. In total, 16.68% (27.10 km^2^) of the region’s area is left out by overlaying the exclusionary layers.

#### Overall FRI and GI site prioritization

**Fig. 8:**
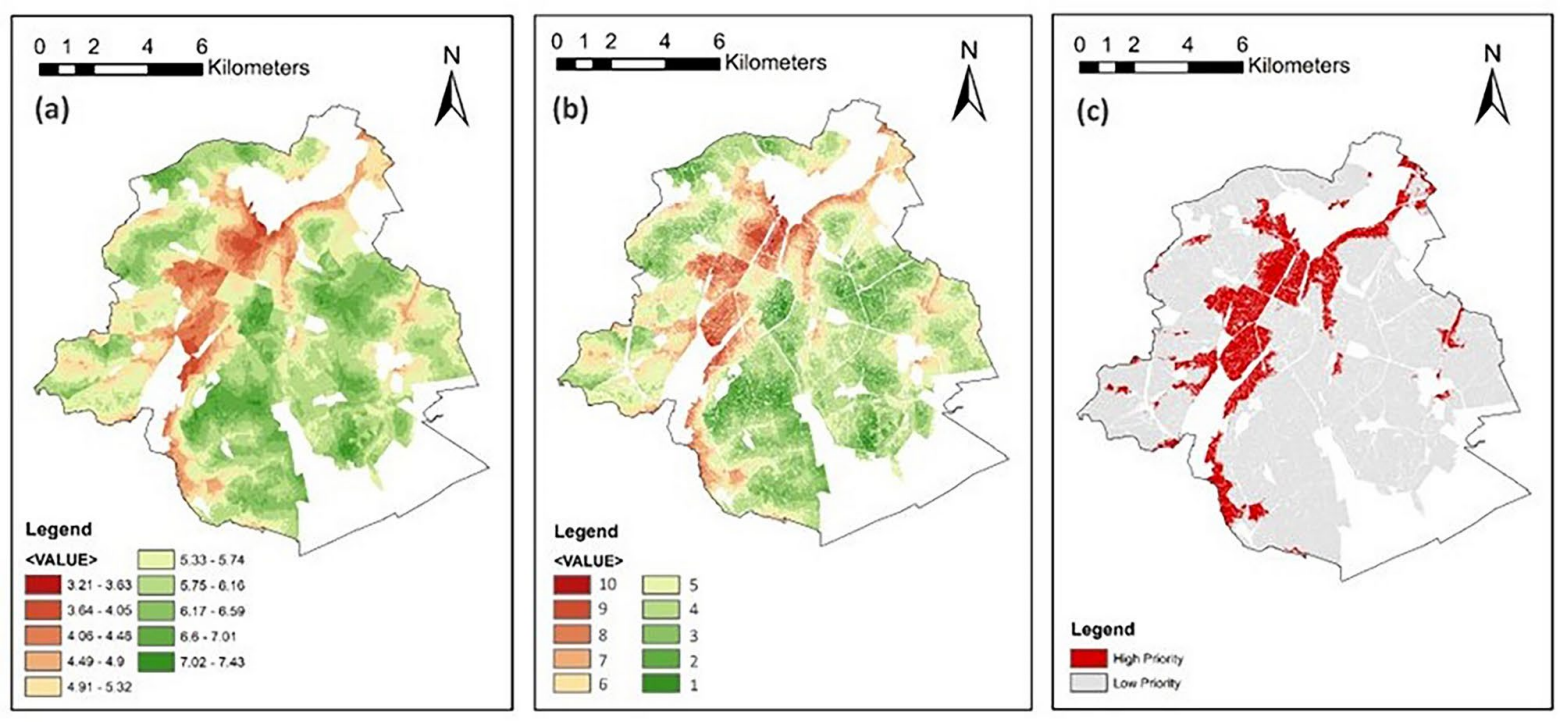
BCR: (**a**) Overall flood resilience index; (**b**) Overall GI implementation priority index; (**c**) Areas with more/less than moderate flood resilience index (high priority: FRI < 5). Maps created by ArcMap 10.8.1^83^.

Eventually, by combining all the generated layers and considering their different weightings, the final outcomes of the FRUGISP model for BCR were generated, dedicating FRI to a total area of 119.46 km^2^ (Fig. 8a). FRI values range between 3.21 and 7.43, highlighting the lowest flood resilience levels in a northeast–southwest strip with a concentration on central consolidated parts. Likewise, areas located near the waterway in Woluwe-Saint-Lambert municipality (eastern side) are categorized as less resilient places. Reclassifying the overall FRI layer and eliminating the unavailable lands, Fig. 8b highlights the places with higher priorities for GI implementations. In total, 101.69 km^2^ was classified into 10 priority levels, with 10 as the highest priority. Locations where the flood resilience level was below the moderate level (FRI < 5) are depicted in Fig. 8c, as high-priority areas for localization of GI. Overall, 15.62% (15.88 km^2^) of the whole analyzed region was labeled high priority, in comparison to 84.38% (85.81 km^2^) of low priority spaces.

## Discussions

The model examines relevant indications of resilience in order to mitigate the potential hazards associated with a flood phenomenon. The prioritizing scheme has significance as it serves as a valuable tool for directing efforts and allocating resources effectively, ensuring that they are directed towards both suitable and prioritized areas with the most need. This can lead to improved management of public funds, a reduction in the negative consequences of urban floods, and therefore enhancing urban resilience through GI applications.

Analyzing fine spatial resolutions of 15 and 20 m (as opposed to the sub-watershed level as demonstrated in studies such as^21^) allows the approach to effectively identify small parcels that are recommended or not suitable for GI implementation, providing a satisfactory level of detail. The utilization of a GIS-based multi-criteria strategy for overlaying grid layers is widely recognized as a prevalent approach for examining several criteria within a geographic context. It has been used by a number of authors to evaluate the suitability of a site for GI implementation, proving its reliability^85,86^. According to recommendations made by Kuller, et al.^25^ for enhancing planning support systems through integrating GI for stormwater management, we have included biophysical and socioeconomic elements in our scheme for GI implementation and site prioritization.

Resilience encompasses the comprehensive assessment of natural hazards throughout their whole cycle^24^, including proactive actions that may be implemented prior to flooding events such as the establishment of proper spatial configurations for urban land use and the evaluation of population density. The implementation of efficient response strategies, such as ensuring the availability of accessible roadways and health amenities, may significantly enhance the expediency and efficiency of immediate rescue actions and emergency aid initiatives. Recovery strategies, such as economic capacities, also facilitate the expeditious restoration of cities to their pre-flood state. The features of the entire flood occurrence process are taken into account in this study, and the chosen evaluation index is based on prior methodologies for assessing flood resilience^6,24,38,39^ and the suitability of GI^17,18^, demonstrating a high level of certainty. However, there is currently no single index or evaluation method to assess urban flood resilience or define GI suitability in addressing water challenges. Future investigations can progressively develop unified frameworks for evaluating these phenomena.

The FRUGISP model successfully identified several locations within the case-study areas that are considered high-priority sites for the installation of GI based on various criteria to enhance flood resilience. For instance, according to the model results for MTY, most high-priority parcels are placed mostly in two sections (see Fig. 5c), indicating explicit suitable starting points for the prioritizing of GI localization efforts. This involves a section in the north (North delegation) characterized specifically by very low socio-economic resilience, and one in the south (Huajuco delegation) categorized mainly with very low infrastructural/environmental resilience.

According to the Urban Development Plan of the Municipality of Monterrey, the North delegation is recognized by the coexistence of formal and informal settlements, the presence of vacant lots (e.g., 25.21% in the San Bernabé zone), shortage of pavement and essential basic services, and a significant deficit of green spaces, except the ecological reserves (i.e., Cerro del Topo Chico area) which are crucial since they offer green spaces that serve as the city’s lungs. Also, the North delegation’s urban image has to be improved since there aren’t enough landmarks or interaction points in the region, and in general, the delegation needs efforts that facilitate its consolidation, improve certain areas, and organize its development^50^.

The situation for the Huajuco delegation is not very different. According to the plan, the zone is bounded to the west by the Cumbres National Park and to the east by the Sierra Cerro de la Silla. Within the delegation, particularly in the upper regions of Cerro de la Silla, there are informal settlements, and some locations lack basic services and pavement. It is crucial to acknowledge that this delegation, which originated from the establishment of country residences, is through a developmental phase characterized by a significant proportion of vacant lots (56.53%). Typically, the delegation necessitates implementing measures that facilitate systematic expansion and safeguard the natural characteristics that are present in the zone^50^.

Currently, the municipality has recovery and improvement targets for both of these categories. Associated with flood risks, the municipality requires “an effective system of conduction and control of rainwater that prevents flooding in overpasses and low areas, as well as the damage caused by runoff, with the dragging of materials and sediments, in urban areas adjacent to the main mountainous elevations^50^”. It includes both delegations. GI implementation can decrease urban runoff through infiltration and retention/detention solutions. On the other hand, it may support the encouragement of the conservation of current natural landscapes and areas that have high values for biodiversity but are adjacent to urban areas and subsequently, endangered by urban activity.

GI implementation also improves the general urban image and decreases the overall deficit of green space, which is a challenge for the whole city and the metropolitan area. According to Carpio, et al.^87^, the metropolitan area lost 28,393 hectares of natural vegetation between 1990 and 2019 as a result of urban development and population increase (e.g., the Huajuco delegation urban development is mostly shaped since 2010), making the situation worse. GI localization also helps decrease carbon emissions, which is specifically a challenge for the North delegation^87^. Vacant lots and abandoned structures are likely to be exploited to offer space for either temporary or long-term usage in GI localization^17^. In general, the zones may boost their urban image, enrich their environment, and raise the population’s life quality through incorporating GI.

For BCR, the high-priority parcels are mainly stretched along the Brussels Canal axis and are mostly located in Brussels, Anderlecht, Forest, and Uccle municipalities. Most parts are situated in central metropolitan locations that serve as the region’s hubs. The area is distinguished by the presence of various services and areas of interest. The southwestern part of the Brussels Pentagon, as the historical city center of Brussels, is also among the assessed high-priority areas.

The priority area’s urban morphology is characterized by a substantial amount of compact and dense structures, a high proportion of impermeable surfaces, little natural vegetation, and low soil infiltration rates. It may be feasible to localize GI systems in places like existing open/green areas, walkways, and public spaces^88^, in addition to buildings (e.g., courtyards^89^). The adoption of GI in this region can support the governmen’s plans (such as The Canal Plan^90^) to restore the area’s former significance and enhance its open public space, and environmental/socioeconomic features. Additionally, it can assist the issue of lack of green space in Brussels’ central areas, which is key to achieving equity and environmental justice^91,92^. The constrained availability of land and the unique demographic characteristics of each site typically provide challenges to the implementation of GI in developed and highly dense metropolitan regions, such as BCR^93,94^. It is caused by a conflict of interest in the distribution of spaces for residential buildings, commercial buildings, recreational areas (including green spaces), infrastructure for transportation, and parking lots^95^.

The lack of available land also limits the amount of surface green space in highly dense urban areas^96^. Consequently, the adoption of small-scale GI is suggested by previous studies. Specifically, these typologies are among the most investigated GI systems that have recently been studied in the field of GI and urban flood resilience^8^. Previous research has indicated that the implementation of small-scale systems can result in a reduction in runoff ranging from 30 to 65% for permeable pavements, up to 100% for rain gardens, and up to 56% for infiltration trenches^97^. Also, a brief period of intense rainfall, lasting around 30 min, has the potential to be fully absorbed by a dry green roof^98^. Besides, the implementation of green roofs can result in a reduction in the volume of runoff by up to 70% and a decrease in the peak flow volume by up to 96%^97^. These methods do not necessitate the acquisition of land from public thoroughfares, therefore rendering their implementation far more feasible^98^. Likewise, GI implementation in building sites (e.g., green roofs and rain gardens) would contribute to the overall sustainability of buildings and the built environment which is especially important in dense urban areas^99–102^. As another consideration, hybrid green-gray solutions can also be applied when space is a limitation^103^.

As land availability is higher in MTY—since the urban development is not dense in comparison to BCR—more GI typologies (small, medium, or big scales) could be suggested to be used in highly prioritized sites. For instance, swales could be implemented in low-density urban areas, which serve as conveyance systems that help to postpone runoff peaks, during intense rain events^104^. Nevertheless, the implementation of vegetation-based GI systems is more restricted by climatic conditions in MTY than in BCR (semi-arid vs. humid climate). Accordingly, non-vegetated GI types (e.g., permeable pavement, water storage tanks, and ponds), or vegetated typologies that are suitable for semi-arid conditions (e.g., using native plants in GI systems) could be advised. Currently, most vegetation types used in the city are not native^50^, so using native species could benefit the whole water cycle in a region with serious water challenges.

As this study has shown, there are spatial synergies and tradeoffs between and across various factors when it comes to localizing GI in order to attain urban flood resilience. Understanding, disputing, and negotiating these tradeoffs and synergies is crucial for enhancing the green infrastructure design process, which has a broad impact on resource utilization, resilience, and quality of life. Implementing a comprehensive and coordinated approach might effectively address the provision of different resilience requirements to places with the greatest needs. The FRUGISP model offers an adaptable tool for facilitating this procedure by implementing a flood resilience-oriented green infrastructure strategy that “seeks to steer spatial planning towards integrated land use governance^105^”.

Although we have shown the critical locations in study areas for GI implementations based on several factors, considering the costs of implementation would be critical for the actual GI practices in the specified locations. Nonetheless, due to the substantial variability in implementation costs influenced by several factors such as the type of GI, project scale, local material and labor expenses, and geographic and urban context, their inclusion in our city-scale studies proved unfeasible. Although some general GI cost estimates exist in certain resources (e.g.^106^), precise evaluation of these costs necessitates comprehensive assessments grounded on local data and conditions, which was beyond the scope of this study. For instance, the construction of swales, bio-retention areas, or green roofs necessitates varying amounts of investment based on several factors, including design criteria, availability of land and materials, and the integration of GI with existing infrastructures.

Nevertheless, what is important to notice is that both research and practice indicate that GI is consistently more cost-effective than conventional gray options when evaluating long-term advantages, such as climate resilience and co-benefits, like biodiversity and air quality enhancement. For instance, a recent review of over 20,000 scientific publications^107^ discovered that GI was consistently found to be cost-effective (in more than 70% of the reviewed studies) in mitigating a wide spectrum of natural catastrophes, such as flooding. This renders GI a highly pragmatic and efficient alternative for urban areas pursuing sustainable flood control strategies.

Considering our study areas, both BCR and MTY have developed plans and policies aimed at supporting climate mitigation and adaptation through GI, supported by the necessary financial resources for implementation. A recent study analyzing the major plans influencing urban planning, water security, and GI implementation in these cities indicates that while a broader array of proposals for water security enhancements utilizing GI is present in BCR, MTY has also initiated the process and is advancing GI implementation through urban policies and initiatives^28^ (e.g., the urban (re)forestation/plantation program by the Metropolitan Environmental Fund of Monterrey^108^). More related to financing, in BCR, while GI mainly comes within the budgetary domain of “Brussels Environment^109^” (in the framework of their approved “Plan Nature^110^” and green space management division), there are also cross-agency/municipality and cross-ministerial budgets that feed into the implementation of GI, such as urban planning and development agency budgets. Likewise, in MTY, most political and financial activities regarding GI development come from the Monterrey Government’s “Secretariat for Sustainable Urban Development^111^” and “Secretariat of Sustainable Infrastructure^112^”, through actions such as *Dirección para Una Ciudad Verde* (Direction for a Green City). Additionally, the Metropolitan Environmental Fund of Monterrey^113^ provides financial mechanisms for GI development within the region.

Our study contributes to the aforementioned efforts in BCR and MTY by pinpointing city-level priority areas where GI interventions can yield optimal resilience, thereby establishing a strategic basis for future, more specific analyses at the neighborhood or site level. These localized studies can leverage our findings to assess and contrast specific GI types in terms of their costs, advantages, and effectiveness in mitigating flooding hazards. Such evaluations are crucial prior to actual GI installations to guarantee the selection of the most profitable and economically feasible solutions.

Given their current frameworks, past achievements, and continued dedication to climate adaptation, BCR and MTY are well-positioned to use our results as a blueprint to advance their efforts to optimize flood resilience through targeted GI interventions. Nevertheless, since our model’s parameters are not location-specific and the model does not need rare or detailed data, the evaluation may be applied to any other city. This research considered 23 indicators in total (19 linked to flood resilience and four related to GI suitability), however, the modeling method supports extra criteria. The model has the potential to be used as a planning tool for applying multifunctional GI in future planning and to help decision-makers decide where to put GI projects so that benefits get maximized.

## Conclusions

The FRUGISP model is developed in this research, making use of a multi-criteria assessment framework, to determine which urban regions should be given the highest priority for the localization of GI systems in order to improve urban flood resistance. We used FRUGISP to analyze the GI site priority in the cities of MTY, Mexico, and BCR, Belgium, which have a variety of distinguishing characteristics but are both impacted by urban floods. The findings highlight the high-priority areas based on the flood resilience index and the availability of land to install GI. We believe that the model can also be utilized for the analysis of other urban regions that face difficulties related to flooding. As a result, decision-makers can use the model to guide future planning activities to identify the most priority sites to locate GI projects to address the difficulties associated with urban floods.

The model and its findings lead to some significant conclusions. First of all, the model pinpoints possible locations for GI implementation as a ‘part’ of a city’s or region’s master or vision plan. So, a more in-depth investigation of prioritized spots with a finer operative resolution is needed in future examinations. Secondly, although our sample size used for AHP ranking is in line with several previous recent studies, the possible impact of this sample size should be noticed when interpreting the outcomes of the exploratory part of our research. As a third point, setting suitable and straightforward priority targets in the final stage (implementation/site level) is crucial for getting the best possible outcomes and providing the greatest potential advantages to the region.

Furthermore, the FRUGISP model is not meant to be used as a basis for selecting individual green infrastructure technologies; for that, a wide range of other considerations are necessary. For instance, filtration techniques are suitable exclusively for regions where groundwater remains uncontaminated^15^. These issues are suggested to be further investigated. Also, considering future climate changes could be applied in selecting both placement sites and GI types^18^, which opens another window for future research.

## Supplementary Information

Supplementary Information.

## Data Availability

All raw data utilized in our analysis was acquired from open databases, except the BCR soil map shapefile that was acquired from the BONAT project team^76^ (see Table 4 for more information). The GIS data used to support the findings could be shared upon request from the corresponding authors.
