# Supplementary material for: Green infrastructure site prioritization to improve urban flood resilience in Monterrey and Brussels using a decision support model
Source: Sci Rep. 2025 Mar 28;15:10744. doi: 10.1038/s41598-025-94851-z (PMC11953299; doi:10.1038/s41598-025-94851-z)
Supplement: Supplementary file 1 — Supplementary Information. [file 41598_2025_94851_MOESM1_ESM.pdf]

Supplementary Material

1. Flood Resilience Sector Maps

1.1. Monterrey

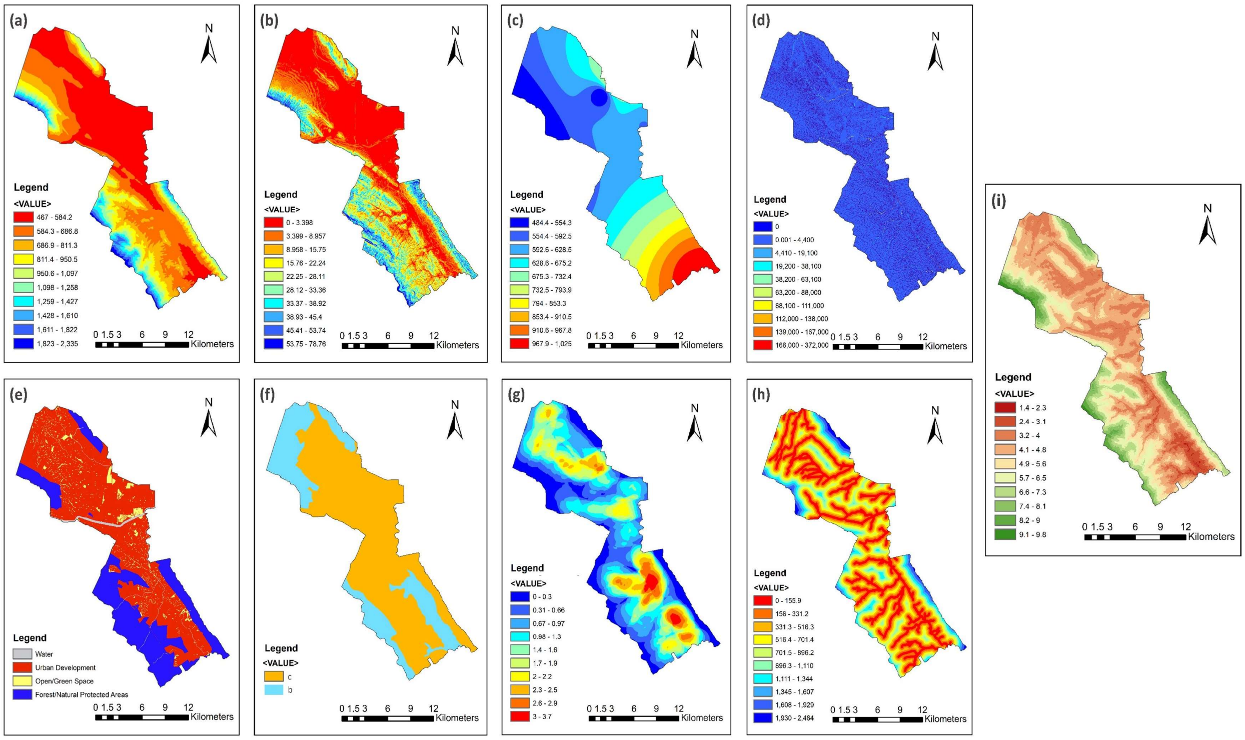

Figure S1. MTY: (a) Elevation; (b) Slope; (c) Annual rainfall intensity; (d) Flow accumulation; (e) LULC; (f) Texture class of soil; (g) Drainage density; (h) Distance to streams; (i) Overall environmental resilience index

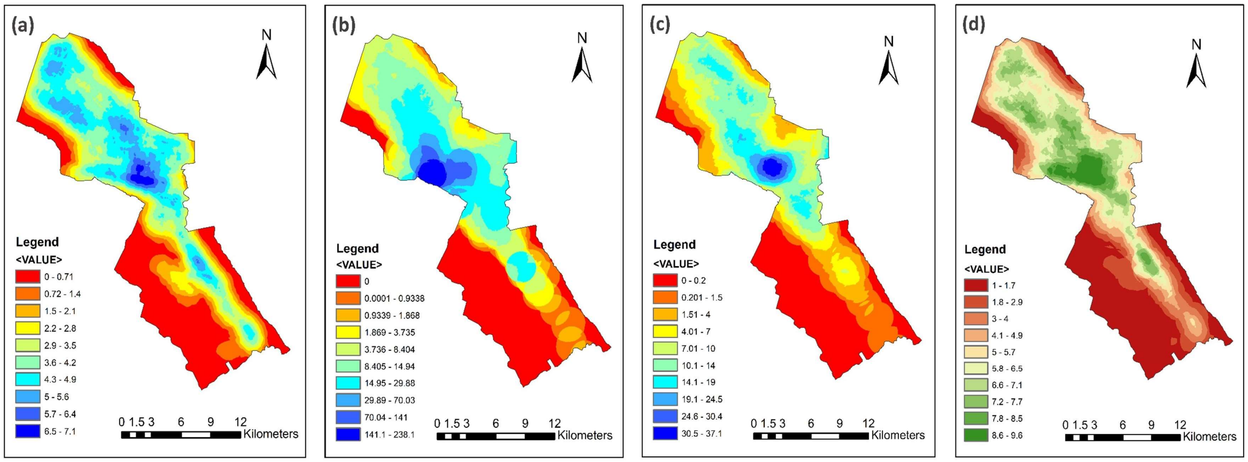

Figure S2. MTY: (a) Road density; (b) Health facilities density; (c) Educational facilities density; (d) Overall infrastructural resilience index

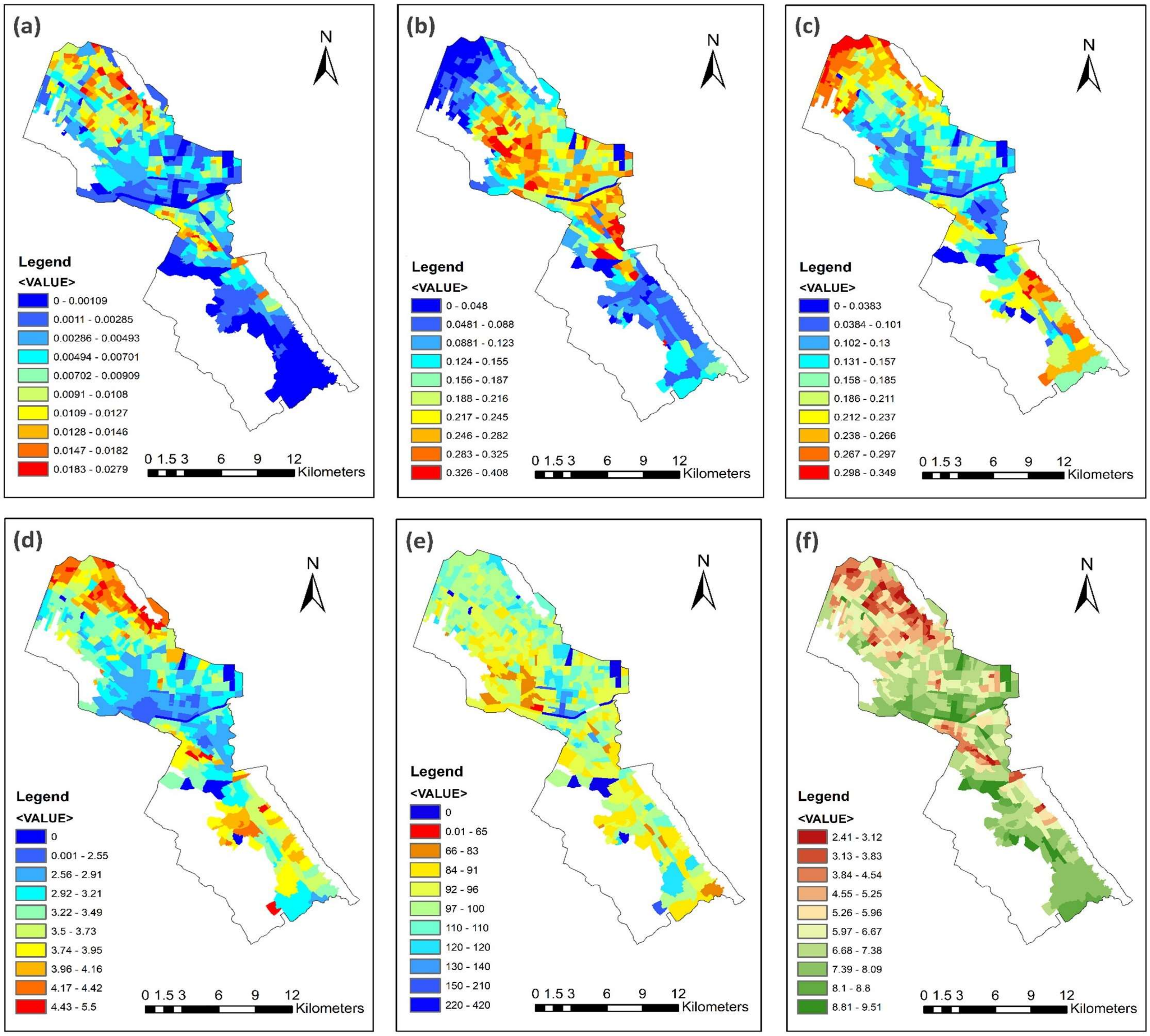

Figure S3. MTY: (a) Population density; (b) Aged pop. rate; (c) Minor pop. rate; (d) Av. household size; (e) Sex ratio; (f) Overall social resilience index

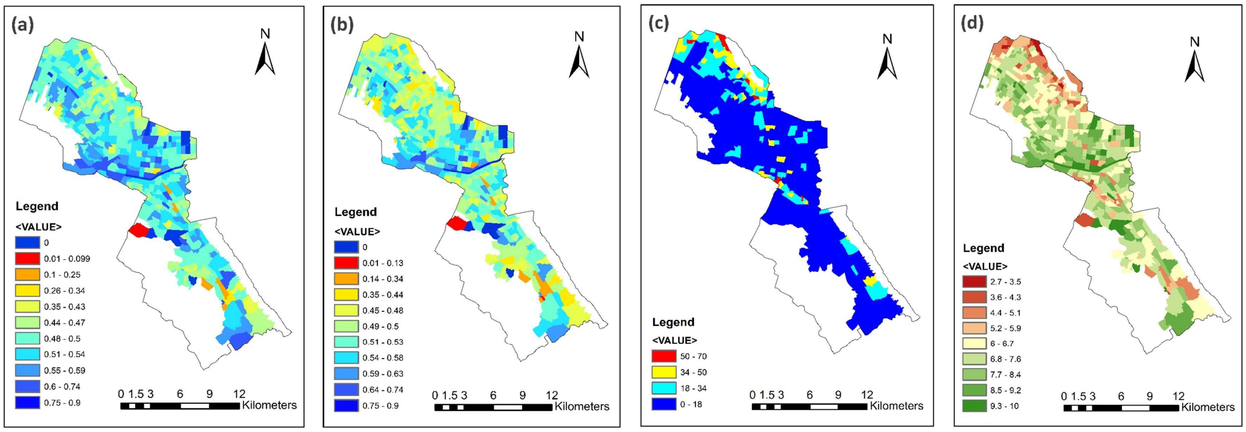

Figure S4. MTY: (a) Employment rate; (b) Economic activity rate; (c) Poverty rate; (d) Overall economic resilience index

1.2. Brussels-Capital Region

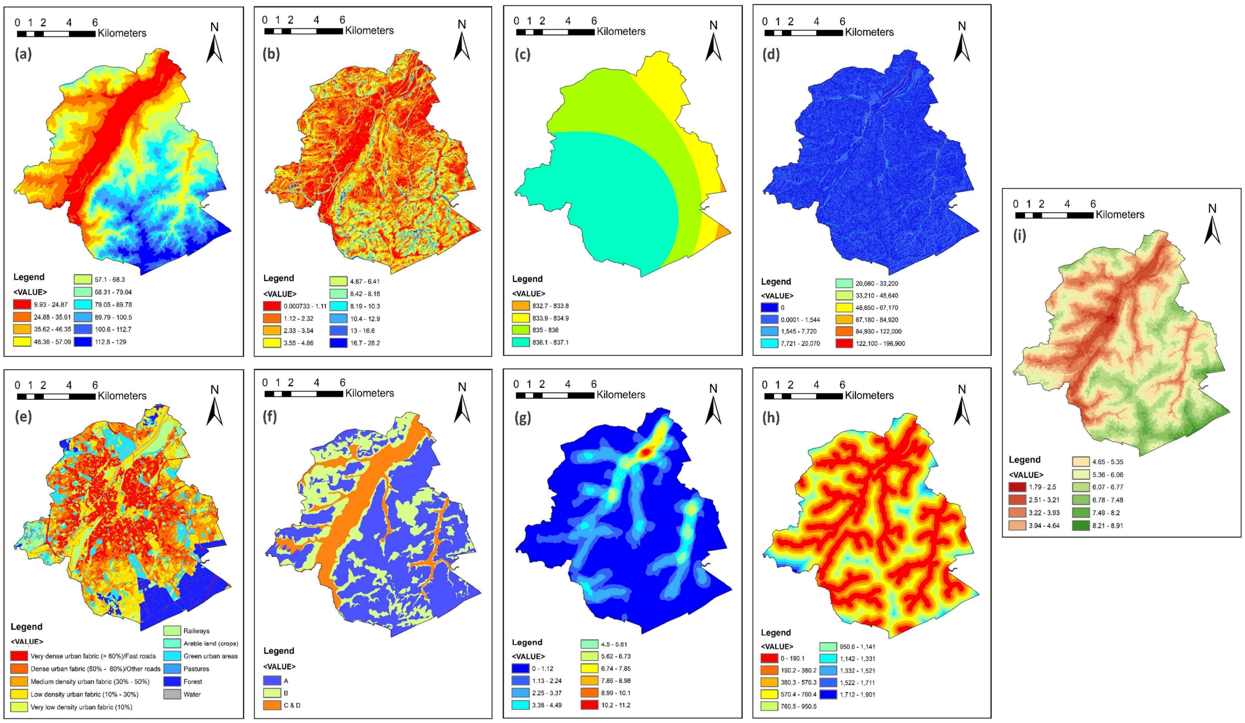

Figure S5. BCR: (a) Elevation; (b) Slope; (c) Annual rainfall intensity; (d) Flow accumulation; (e) LULC; (f) Hydrologic class of soil; (g) Drainage density; (h) Distance to streams; (i) Overall environmental resilience index

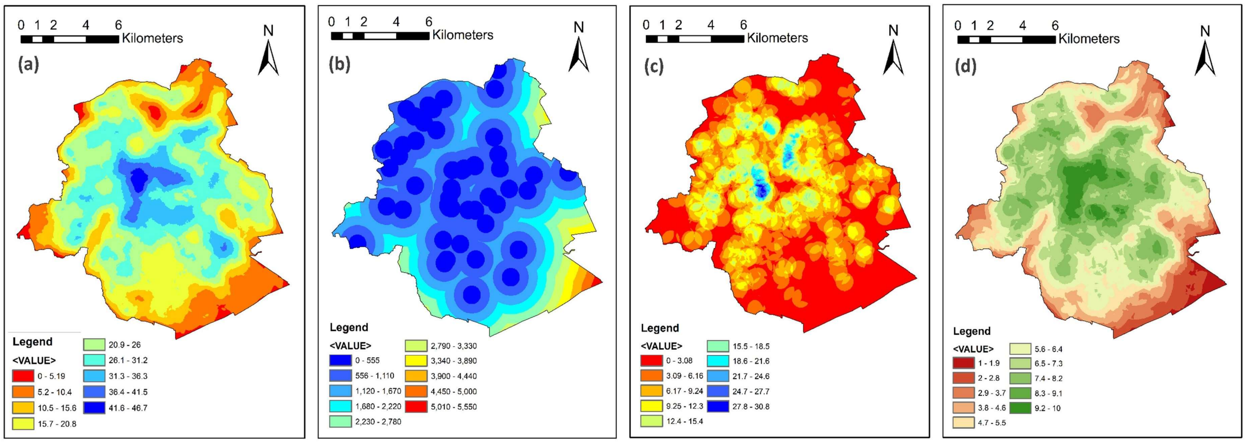

Figure S6. BCR: (a) Road density; (b) Distance to hospitals; (c) Educational facilities density; (d) Overall infrastructural resilience index

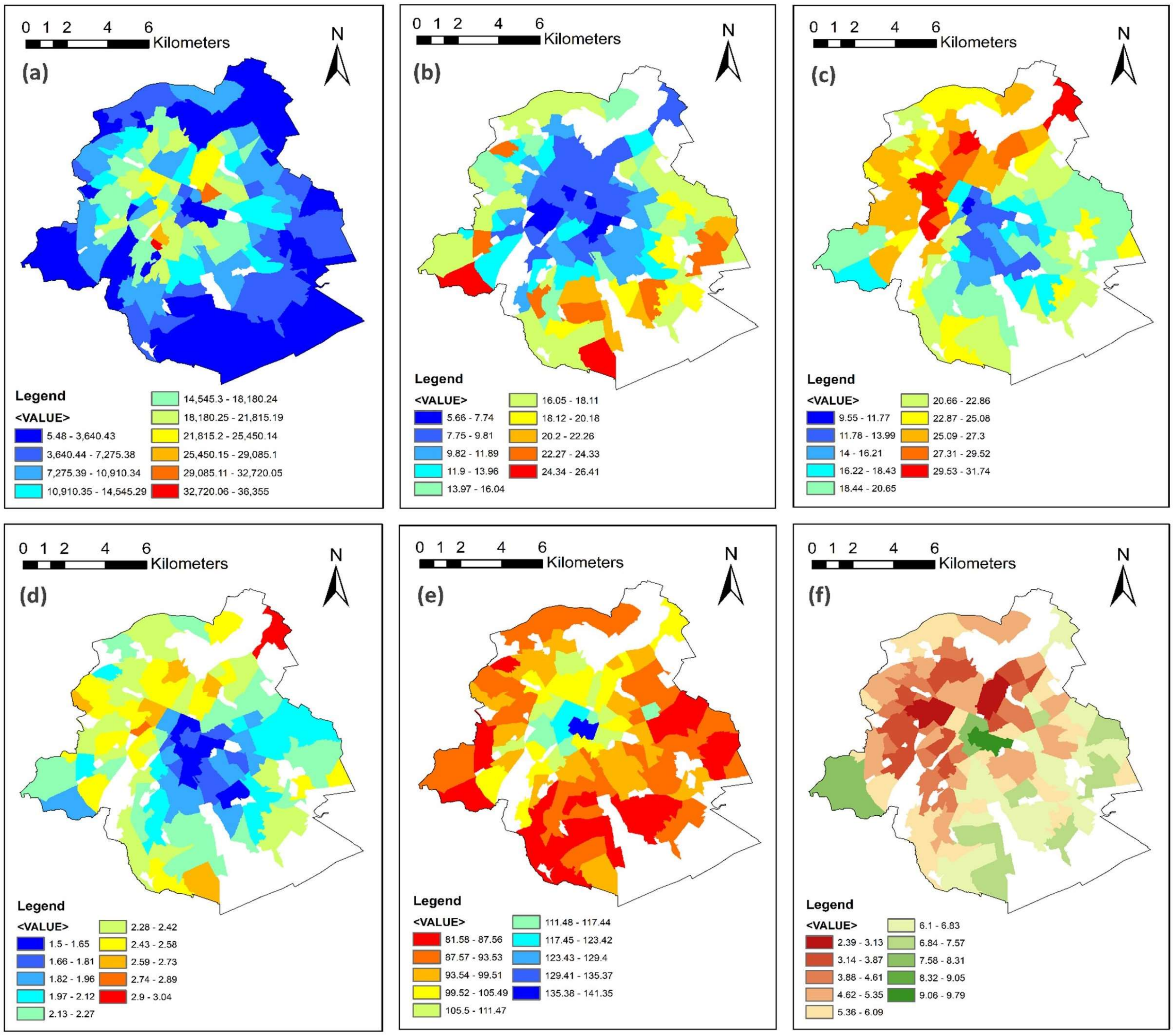

Figure S7. BCR: (a) Population density; (b) Aged pop. Rate; (c) Minor pop. rate; (d) Av. household size; (e) Sex ratio; (f) Overall social resilience index

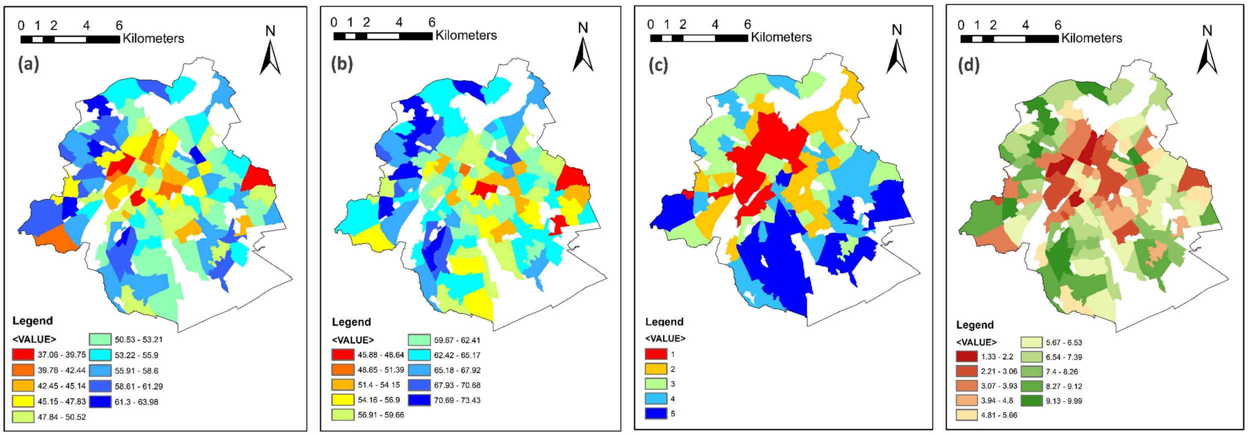

Figure S8. BCR: (a) Employment rate; (b) Economic activity rate; (c) Average income class; (d) Overall economic resilience index

2. Index Indicator Weights, Experts Profiles, and Pairwise Evaluation Matrices

Table S1 shows the assigned weightings to the FRI indicators and sectors. Five different judgment matrices were built to assign the weightings (Tables S4-S8), one to evaluate the prioritization of the four resilience sectors and the other four tables to assess the intra-sector weightings of the indicators. The judgments were based on Saaty's defined significance scales and their interpretations (Table S2). The profiles of the experts can be seen in Table S3.

Table S1. AHP weightings assigned to the urban flood resilience index indicators and sectors.

Target	Resilience Sector	Sector Weight	Flood Resilience Index Indicator	Intra-Sector Weight	Extra-Sector Weight
Flood Resilience	Environment	0.455	Elevation	0.250	0.114
			Slope	0.113	0.051
			Av. Annual Rainfall Intensity	0.156	0.071
			LULC	0.041	0.019
			Soil Type	0.031	0.014
			Flow Accumulation	0.083	0.038
			Drainage Density	0.103	0.047
			Distance to Streams	0.221	0.101
	Infrastructure	0.263	Road Density	0.575	0.151
			Health Facilities Density/Distance to Hospitals	0.366	0.096
			Educational Facilities Density	0.059	0.015
	Society	0.141	Population Density	0.517	0.073
			Aged Population Percentage	0.127	0.018
			Minor Population Percentage	0.226	0.032
			Av. Household Size	0.080	0.011
			Sex Ratio	0.049	0.007
	Economy	0.141	Av. Income Class/Rate of Poverty	0.333	0.047
			Employment Rate	0.333	0.047
			Economic Activity Rate	0.333	0.047

Table S2. The judging matrix's relative significance scales and their interpretations.

Significance Scale	Interpretation	Significance Scale	Interpretation
1	Equal importance	6	Strong-to-very strong importance
2	Equal-to-moderate importance	7	Very strong importance
3	Moderate importance	8	Very-to-extremely strong importance
4	Moderate-to-strong importance		
5	Strong importance	9	Extreme importance

Table S3. Characteristics of the experts.

Characteristics	Category	Number
Gender Identity	Male	3
	Female	4
Educational Level	PhD	4
	PhD researcher	3
Profession	Urban water/flood management	3
	Urban planning	2
	Both	2
Self-reported knowledge of urban flood resilience analysis	Limited	0
	Reasonable	3
	Very good	4
Total number of participants		7

Table S4. The AHP pairwise evaluation matrix for the four FRI sectors.

Resilience Sector	Environmental	Social	Economic	Infrastructural
Environmental	1.00	3.00	3.00	2.00
Social	0.33	1.00	1.00	0.50
Economic	0.33	1.00	1.00	0.50
Infrastructural	0.50	2.00	2.00	1.00
Sum	2.17	7.00	7.00	4.00

Table S5. The AHP pairwise evaluation matrix for environmental flood resilience factors.

Indicator	Elevation	Slope	Rainfall	Flow Accumulation	Drainage Density	LULC	Soil Type	Distance to Streams
Elevation	1.00	4.00	5.00	4.00	5.00	5.00	4.00	0.33
Slope	0.25	1.00	3.00	3.00	0.167	3.00	3.00	0.125
Rainfall	0.20	0.33	1.00	2.00	3.00	4.00	4.00	2.00
Flow Accumulation	0.25	0.33	0.50	1.00	1.00	2.00	3.00	1.00
Drainage Density	0.20	6.00	0.33	1.00	1.00	2.00	3.00	0.50
LULC	0.20	0.33	0.25	0.50	0.50	1.00	2.00	0.25
Soil Type	0.25	0.33	0.25	0.33	0.33	0.50	1.00	0.25
Distance to Streams	3.00	8.00	0.50	1.00	2.00	4.00	4.00	1.00
Sum	5.35	20.33	10.83	12.83	13.00	21.50	24.00	5.46

Table S6. The AHP pairwise evaluation matrix for infrastructural flood resilience factors.

Indicator	Road Density	Health Facilities Density/ Distance to Hospitals	Edu. Facilities Density
Road Density	1.00	2.00	8.00
Health Facilities Density/ Distance to Hospitals	0.50	1.00	8.00
Edu. Facilities Density	0.13	0.13	1.00
Sum	1.63	3.13	17.00

Table S7. The AHP pairwise evaluation matrix for social flood resilience factors.

Indicator	Pop. Density	Aged Population	Minor Population	Sex Ratio	Household Size
Pop. Density	1.00	5.00	4.00	7.00	5.00
Aged Population	0.20	1.00	0.50	3.00	2.00
Minor Population	0.25	2.00	1.00	5.00	4.00
Sex Ratio	0.14	0.33	0.20	1.00	0.50
Household Size	0.20	0.50	0.25	2.00	1.00
Sum	1.79	8.83	5.95	18.00	12.50

Table S8. The AHP pairwise evaluation matrix for economic flood resilience factors.

Indicator	Employment Rate	Econ. Activity Rate	Av. Income Class/Poverty Rate
Employment Rate	1.00	1.00	1.00
Econ. Activity Rate	1.00	1.00	1.00
Av. Income Class/Poverty Rate	1.00	1.00	1.00
Sum	3.00	3.00	3.00
